# Sympathetic nerve activity can be estimated from skin conductance responses — A comment on Henderson et al. (2012)^☆^

**DOI:** 10.1016/j.neuroimage.2013.08.030

**Published:** 2014-01-01

**Authors:** Dominik R Bach

**Affiliations:** Wellcome Trust Centre for Neuroimaging, University College London, United Kingdom Berlin School of Mind and Brain, Humboldt-Universität Berlin, Germany

**Keywords:** Skin conductance responses, Skin sympathetic nerve activity, Electrodermal activity, Galvanic skin response, GSR, EDA, SCR, SSNA, Sympathetic arousal, Mathematical model, Biophysical model

## Abstract

A recent paper by Henderson et al. (2012) claimed that skin sympathetic nerve activity (SSNA) can not be retrieved from skin conductance responses (SCR). Here, I argue that this claim is not supported by the literature, and comment on contemporary approaches of estimating SSNA from SCR using biophysical models.

## Highlights

• Sympathetic arousal can be estimated from skin conductance responses (SCR).
• Such estimation relies on estimation of skin sympathetic nerve activity (SSNA).
• Physiological work has established a linear relationship of SCR and SSNA amplitudes.

I read with great interest the paper by Henderson et al. (2012) on the relationship of SSNA and BOLD responses. The generation of the SCR via skin sympathetic nerve fibres remains an understudied topic despite the popularity of SCR as an indicator of sympathetic arousal in emotion neuroscience. However, in order to motivate their use of SSNA for fMRI analysis, Henderson et al. (2012) claim that SSNA cannot be retrieved from SCR. This is not supported by the literature.

The claim mainly rests on a statement taken from Bini et al. (1980): “No simple quantitative relationship could be seen between the size of individual sudomotor bursts and accompanying electrodermal responses”. However, in the original paper, Bini et al. (1980) continue: “This was partly due to the fact that succeeding electrodermal deflexions merged when the sudomotor burst incidence was high, partly due to the non-linear characteristics of the electrodermal apparatus when measuring over a wide range of absolute skin resistance. During the initial phases of thermoregulatory sweating, however, sudomotor burst incidence was low enough to allow a quantitative correlation between the mean voltage amplitude of individual sudomotor bursts and the amplitude of corresponding transient changes of skin resistance. As shown in Fig. 5, the relationship was linear with a considerable scatter.”

This is to say that when accounting for technical artefacts, there was a simple quantitative (i.e. linear) relationship between SSNA and SCR amplitude in the study by Bini et al. (1980). Results from yet another experiment are in keeping with this: The amplitude of SSNA bursts is linearly related to the maximal rate of sweat expulsion; and, somewhat more weakly, to the integrated sweat production during the skin response (Sugenoya et al., 1990).

As a further argument to their claim, Henderson et al. (2012) discuss nerve stimulation studies. Kunimoto et al. (1991, 1992) note a non-linear relation of sudomotor nerve stimulation with the ensuing SCR. As a likely reason, they discuss a non-linear relationship between stimulation and the elicited SSNA. This will of course also lead to a non-linear relation between stimulation and SCR — but they did not control SSNA to account for this possibility.

Finally, Henderson et al. state Kirno et al. (1991) in support of their claim. However, this study was not concerned with the relationship between SSNA amplitude and SCR amplitude at all. Instead, they highlighted non-linearities of overlapping responses when stimulation frequency exceeded 0.5 Hz. This is a stimulation frequency not recommended for SCR research (Boucsein, 2012), and not used by Henderson et al. in their present experiment either.

While Henderson et al. (2012) conclude that “changes in skin conductance cannot be used to quantify changes in SSNA”, the aforementioned literature reveals a picture that is not quite so bleak. Indeed, SCR have been successfully used as an operational index of sympathetic arousal over decades under the assumption that sympathetic arousal is transmitted via SSNA (Boucsein, 2012).

Recent advances in biophysical SCR modelling have contributed to physiological evidence about the relationship of SSNA and SCR. All of these models assume that SSNA and SCR are closely related via a linear time-invariant filter. In one line of research, deterministic SCR models are inverted to yield estimates of SSNA without making assumptions about the form of SSNA (Alexander et al., 2005; Benedek and Kaernbach, 2010a,b): SSNA estimates in these studies show individual compact bursts, similar to neural recordings of actual SSNA. Another family of models makes assumptions about the shape of SSNA bursts and is able to estimate sympathetic arousal from SCR (Bach et al., 2009, 2010a,b, 2011; Bach and Friston, 2013; Bach et al., 2010c): sympathetic arousal estimates in these studies better predict a psychological manipulation than observed SCR.

All these studies lend credence to the assumption that SSNA and SCR can be related by simple biophysical models. This calls for an integrated effort, rather than a somewhat artificial juxtaposition, of SSNA physiologists, and SCR methodologists, to provide more powerful tools to access neural processes by simple observations.
